# Cocreated Smartphone App to Improve the Quality of Life of Adolescents and Young Adults with Cancer (Kræftværket): Protocol for a Quantitative and Qualitative Evaluation

**DOI:** 10.2196/10098

**Published:** 2018-05-10

**Authors:** Abbey Elsbernd, Maiken Hjerming, Camilla Visler, Lisa Lyngsie Hjalgrim, Carsten Utoft Niemann, Kirsten Boisen, Helle Pappot

**Affiliations:** ^1^ School of Medicine University of Kansas Kansas City, KS United States; ^2^ Department of Oncology Rigshospitalet, Copenhagen University Hospital Copenhagen Denmark; ^3^ Department of Hematology Rigshospitalet, Copenhagen University Hospital Copenhagen Denmark; ^4^ Department of Pediatric Hematology and Oncology The Child and Youth Clinic Rigshospitalet, Copenhagen University Hospital Copenhagen Denmark; ^5^ Center for Adolescent Medicine Rigshospitalet, Copenhagen University Hospital Copenhagen Denmark

**Keywords:** AYA, adolescent and young adult, cancer, mHealth, oncology, cocreation, HRQoL, quality of life, smartphone

## Abstract

**Background:**

Adolescents and young adults with cancer face significant challenges during the course of their medical treatment and recovery from illness. Many adolescents and young adults struggle with long-term complications in the physical, psychosocial, economic, and academic domains. Mobile health (mHealth) interventions provide an innovative platform for delivering supportive care, particularly through the utilization of apps on smartphones and tablets. To create a successful mHealth intervention for adolescents and young adults, youth input and feedback is essential. The process of cocreation, in which the target app user has a direct role in dictating design and function, was utilized to create the prototype smartphone app for adolescents and young adults with cancer, “Kræftværket.”

**Objective:**

The objective of this paper is to describe the protocol for the evaluation of the Kræftværket app, a prototype app designed via cocreation, to support and improve health-related quality of life for adolescents and young adults with cancer.

**Methods:**

The Kræftværket app has three primary features, (1) a symptom and activity diary, (2) a supportive communication network between app users, and (3) a “one-stop shop” information bank with practical information as well as links to patient organizations and other resources. The app will be evaluated in two phases, a pilot test and an implementation test. In the pilot test, the app will be launched to a test group of 20 adolescents and young adults aged 15 to 29 years, selected for equal representation amongst age group and treatment status. Patients will be allowed to utilize the app over the course of six weeks and will complete a baseline and follow-up European Organization for Research and Treatment of Cancer Quality of Life Questionnaire Core 30 (EORTC QLQ-C30) health-related quality of life inventory. In addition, participant focus group interviews will be conducted according to a semistructured interview guide. Resulting data will be analyzed using thematic analysis. Results and appropriate analysis from both the qualitative and quantitative branches of the pilot test will be discussed amongst the research group, and appropriate changes based on user feedback will be made to the app before the final project phase. In the implementation test, the app will be provided and utilized by a sample of 50 adolescents and young adults aged 15-29 years selected for equal representation amongst gender, age group, diagnosis, and treatment status over the course of 3 months. Participants will be asked to complete a baseline and follow-up EORTC QLQ-C30 HRQoL inventory.

**Results:**

Pilot testing is expected to take place in February 2018, and implementation testing is expected to begin May 2018.

**Conclusions:**

It is the hope that Kræftværket app will serve as a beneficial and easily utilized product. The process of evaluating the app and its effect on quality of life will address the absence of evidence-based mHealth interventions, and attempt to validate new approaches to benefitting adolescents and young adult oncology patients in the digital world.

**Registered Report Identifier:**

RR1-10.2196/10098

## Introduction

Adolescents and young adults (AYAs) are a group with health care needs which are separate from both adult medicine and pediatrics. Patients in this age group present unique and significant physical, psychological, and cognitive challenges through hospitalization and beyond, which have often been overlooked, particularly in the fields of hematology and oncology [1-5]. AYAs with cancer report a decrease in health-related quality of life (HRQoL), regardless of other demographic factors such as gender, age, or ethnicity [6,7] and struggle with long-term complications in the physical, psychosocial, economic, and academic domains as a consequence of their disease and recovery process [8-10].

While the age range definition for AYA patients varies by organization, the Danish Cancer Society has compiled a large-scale report addressing the AYA oncology population aged 15 to 29 years [5]. This age range is used at our institution, as opposed to those proposed by the WHO (ages 12-24 years) or US National Cancer Institute (ages 15-39 years), in order to complement existing literature on AYA hematology and oncology in Denmark, as well as to correspond with the age range provided for AYA support organizations in Denmark [5,11,12]. Within this age range, approximately 500 AYAs in Denmark are diagnosed with cancer each year [5,13]. While this is a small number of patients, conducting AYA cancer research in Denmark is convenient due to a highly accessible cancer registry and the elimination of certain variables which could influence cancer research, such as health care insurance status, due to the presence of a nationalized health care system [14,15].

Technology provides a contemporary method of delivering health interventions, particularly to AYA patients. Many interventions have previously been designed specifically for AYA cancer patients and contain tools to assist with symptom tracking, health promotion, and social networking [16,17]. Mobile health (mHealth) apps are commonly used to design health interventions and can be highly beneficial due to their ability to portably connect patients to peers, health care teams, and validated sources of information, as well as to complement existing technologies such as Web-based interventions or health tracking devices. These apps can be used on tablets or smartphones, where the term “smartphone” is defined as a mobile phone with additional functionalities, such as internet access and has an operating system capable of downloading and running such apps. Such apps have various purposes, including, but not limited to, social networking, health tracking, health promotion, and provision of information [18,19]. The current availability of mHealth apps for AYAs with cancer is widespread, however, there are limitations to both the content and validity of these apps. Very few apps have been developed with a defined protocol involving both health care professionals and AYAs, and even fewer have demonstrated their effectiveness and benefit to the population [19-21]. It is possible, however, that these tools may harbor potential for improvement of HRQoL in AYA cancer patients.

Additionally, mHealth apps could be of value due to their perceived ability to positively influence self-efficacy, empowerment, and self-management capabilities of patients [22]. Bandura et al have described the theory of self-efficacy, defining self-efficacy as people’s beliefs about their ability to influence change throughout their lives [23]. Frequently, self-efficacy is described in coordination with empowerment, linking the individual’s personal abilities with greater structures in political and social domains [24,25]. High levels of self-efficacy and empowerment provide individuals a sense of personal agency and ability to exercise control of their surroundings [23-28]. This may be reflected in improved abilities of self-management, or an individual’s ability to manage the consequences of living with disease on a physical, social, and psychological level [25-29].

Therefore, in order to create a successful mHealth intervention, the input of AYAs themselves is critical to both the design and evaluation of such interventions. Many existing technologies intended for patient use are not developed or evaluated based on user perspectives and, as such, not all existing mHealth interventions intended for AYAs are user-friendly or adequately meet the needs of their target populations [20,21,30,31]. AYA populations are frequent consumers of mobile technology and would therefore benefit from technology resources developed with their perspective [5,32-34].

At a technology intervention idea workshop, AYAs were asked to discuss their life with cancer, identify challenges they faced, and then discuss a plan for a technology-based intervention to address these needs. The perspective of both AYAs currently receiving treatment for cancer and survivors in remission was requested. At the end of the workshop, the participants concluded that a smartphone app would be an effective tool for empowering AYAs and improving their HRQoL [35]. Based on this idea, funding was raised and the process of cocreating an app, involving AYAs with cancer, was initiated to develop a prototype for a user-friendly smartphone app. The current app prototype has been designed to serve as a support tool for AYAs, integrating community support features, symptom tracking, and a “one-stop shop” information database into one’s pocket for maximal convenience and benefit as a cancer patient or survivor.

Based upon the background of mHealth interventions for AYAs and our developed app prototype, the objective of this article is to describe the protocol for the evaluation of the Kræftværket app, a smartphone app designed via cocreation for AYAs with cancer.

## Methods

### Participants and Recruitment

Kræftværket is a youth support center and social organization for AYAs with cancer aged 15 to 29 years at Rigshospitalet in Copenhagen, Denmark [36]. The name of both this center and the smartphone app described in this project, Kræftværket, is composed of the Danish words for “power plant” (Kraftværk) and “cancer” (Kræft), evoking empowerment throughout the time of cancer treatment and recovery. Throughout all phases of the project, patients will be recruited from a population of AYAs who are currently receiving or have received treatment at Rigshospitalet for cancer. Participants will be invited to participate in the study by a youth coordinator either by physical meeting or via the closed Kræftværket Facebook group. The youth coordinator will explain participation details and the risks and benefits of participation. Eligible participants who are already part of the closed Facebook group will be provided these details via a personal message. Eligible participants who are not already part of the Facebook group will be invited to join but will not be required to do so.

Inclusion criteria from this population will be AYAs aged 15 to 29 years with access to smartphones and the internet, including cellular data or Wi-Fi. Exclusion criteria will be those with an inability to read and write in Danish. Recruitment will be targeted to include a broad range of participants across the AYA spectrum with diversity in age (groups of participants aged 15-22 years or ≥23 years), gender, diagnosis, and treatment status (receiving active treatment or not receiving active treatment). Throughout phases II and III, participants that represent these demographics will be approached and targeted. Targeted recruitment for participants in Phase II will be based on age group and recruitment status, while in Phase III targeted recruitment will be based on gender, age group, treatment status and diagnosis. Sample sizes for phase II and III were determined based on prior research from other mHealth interventions [37,38].

### Primary Intervention and Kræftværket App Features

**Figure 1:**
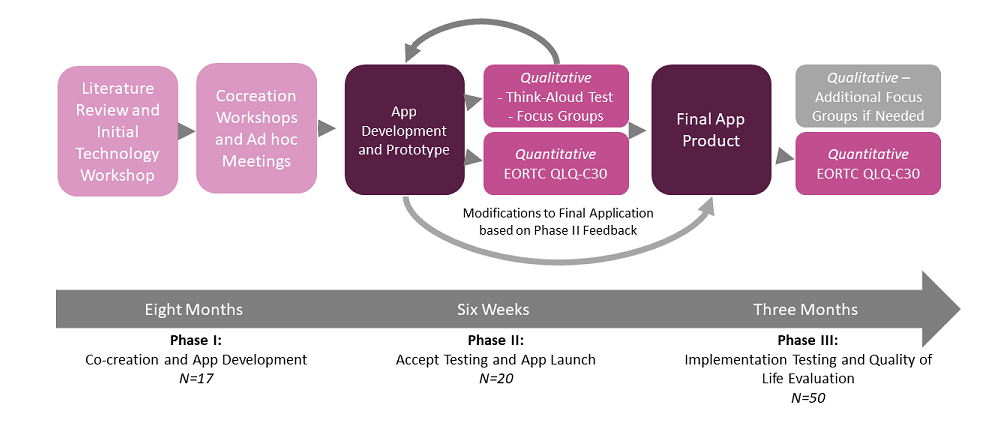
Framework for development of Kræftværket smartphone app and evaluation of study design. EORTC QLQ-C30: European Organization for Research and Treatment of Cancer Quality of life Questionnaire Core 30.

The primary intervention is the smartphone app Kræftværket (named after the aforementioned youth sanctuary at Rigshospitalet), a comprehensive smartphone app designed for AYAs with cancer and AYA cancer survivors. After the research team performed a primary review of the literature, an idea generation workshop was performed in coordination with 12 AYAs attending Kræftværket to identify, create, and evaluate a single technology intervention that could increase self-empowerment and improve quality of life (QoL) in AYAs with cancer. This workshop confirmed that a smartphone app was an appropriate tool for intervention. The technology intervention workshop was initiated and run by the Kræftværket research group and it was specific for the population of AYAs with cancer. A cocreation project was initiated to develop the smartphone app designed from the input of AYAs. The initial workshop and all subsequent workshops were held outside of the hospital and were run by members of the research team or representatives from the partnering technology developer. Further project details, explanation of the cocreation process, and details about the app prototype are provided in a separate paper [39]. Phases II and III will evaluate the design model, allowing for changes based on the obtained results and feedback (Figure 1).

Cocreation is one method of tool development with patient and public involvement (PPI). PPI is believed to increase both the quality and relevance of a given product to its target population in terms of research objectives and outcomes [40]. As such, PPI is of frequent interest in social science and health care research.

Cocreation is a process that allows the individuals who will utilize a product to have an active voice in its development, therefore shifting the focus and involvement away from professional stakeholders (such as health care professionals and the research team), toward those who would use the final product [39]. The process of cocreation was specifically selected to create a user-friendly app that will be beneficial and enjoyable for the target patient population [31,32,41]. During each workshop, the goals for the app were prioritized based on the AYA patients’ specifications. After different concepts were developed, feedback was requested from the AYAs and the feedback was then integrated into the app. This process continued iteratively, where feedback would again be requested and integrated into the app, and this process would continue until majority agreement was achieved on the final product. The app prototype was finalized after a series of three cocreation workshops with the input of 17 AYAs in total [39].

**Figure 2:**
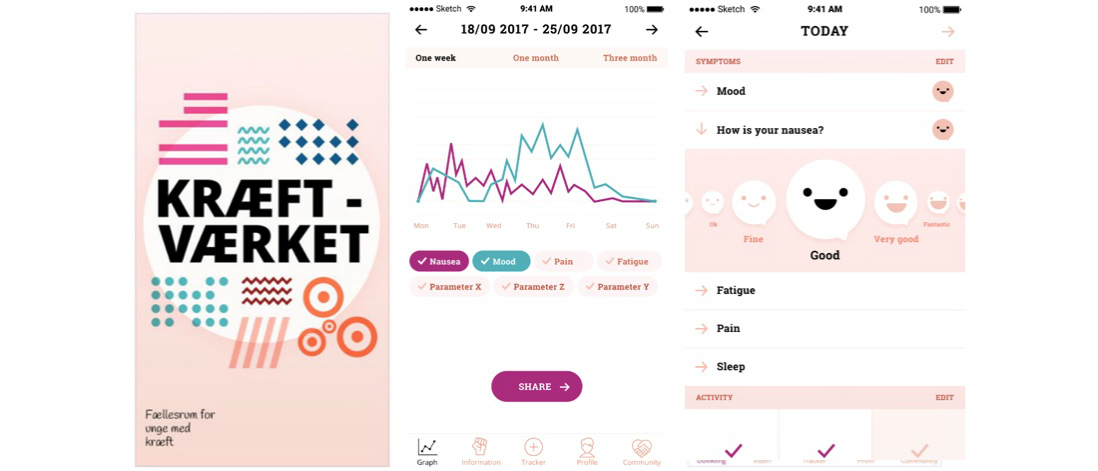
Screenshots of current development model for Kræftværket app.

The Kræftværket app is a tool designed to be utilized both during and after cancer treatment and will be available on both iOS and Android platforms. Initial feedback from AYA patients at the first cocreation workshop identified three essential features of the smartphone app (Figure 2). These included (1) a diary which allows AYAs to track how disease and treatment may affect their daily life and mood which will be demonstrated visually as an insight-graph for patients to track their physical, mental, and emotional status; (2) a communication network between app users to share knowledge and support with one another at any time, in the form of direct messaging and a public forum and (3) a “one-stop shop” information bank, where AYAs can access practical information, useful references, and links to patient organizations and other resources. The specific features of the Kræftværket app are presented in Textbox 1. Further details of the cocreation process, as well as its utilization in the development of Kræftværket app, and the app prototype’s features are described in greater detail in a separate publication [39].

The functional prototype of the Kræftværket app will be launched in February 2018 for analysis in Phases II and III of the mHealth intervention project. Table 1 presents an overview of the target participants, data collection and analysis methods for phases II and III.

Both the proposed development and analysis of the Kræftværket app are intended to follow the Model for Assessment of Telemedicine Applications (MAST) framework for assessing the effectiveness and contribution quality of telemedicine apps based on scientific data. The MAST framework defines a 3-level approach to evaluating eHealth interventions addressing: (1) preceding considerations for intervention purpose and specificities, (2) assessments within 7 domains (the health problem and characteristic of the app, safety, clinical effectiveness, patient perspectives, economic aspects, organizational aspects, and sociocultural, ethical, and legal aspects), and (3) transferability of the intervention to areas of expansion [42,43]. Table 2 summarizes specific outcome measurement goals and considerations taken during the development process of Kræftværket in relation to the MAST framework.

### Phase II: Pilot Testing

The app will be launched to a test group of 20 AYAs. In the test group, 10 patients will be currently receiving treatment and 10 patients will have completed cancer treatment. Recruitment in both patient groups will be targeted for an appropriate representation of gender and age groups across the AYA spectrum (ages 15-22 and 23-29 years). During the pilot test, patients will utilize the app over the course of 6 weeks. During both phases II and III, patients will not be given any specific instructions on the frequency that they should use the app. They will be instructed to use the app as they see fit. Patients will provide baseline measurements of QoL using the European Organization for Research and Treatment of Cancer Quality of life Questionnaire Core 30 (EORTC QLQ-C30), a validated, internationally recognized tool for scoring quality of life among cancer patients [44]. At this time, HRQoL is the only outcome investigated by this protocol. No other EORTC tools or modules will be used in this protocol. At the end of the 6-week period, the participants will be prompted to complete the EORTC QLQ-C30 via the app. Quantitative EORTC QLQ-C30 data will be analyzed using one-way analysis of variance (ANOVA) test.

**Description of Kræftværket app features. AYA: Adolescents and young adults.**

**Symptom and Activity Diary**

- Customizable diary to track and rate symptoms and wellness on a 5-point visual scale.
- Diary includes suggested tracking metrics, such as energy, nausea, or pain, as well as ability for users to add desired personal metrics.
- Checkboxes are included for daily activities, such as taking medication, meditating, or exercising.
- Ability to view prior entries on a 1-week, 1-month, or 3-month timeline and toggle metrics to view metrics altogether, or separately.

**Communication Network and Forum**

- Community forum for registered app users to post and share ideas and photos with other users under specific grouped topics.
- Anonymous posting feature.
- Private messaging function.

**Information Bank**

- Verified AYA-specific information from Danish Cancer Society.
- Videos featuring AYA cancer patients and survivors giving testimony on personal experience.
- Outgoing links from app to other patient support organizations and resources.
- Link to YouTube channel for uploading personal videos and testimony.

**Table 1:** Overview of Phase II and Phase III participants, data collection methods, and data analysis methods. ANOVA: analysis of variance; EORTC QLQ-C30: European Organization for Research and Treatment of Cancer Quality of life Questionnaire Core 30.

Project Phase		Data Collection	Data Analysis
**Phase II: Pilot Testing, N=20**			
	Quantitative	EORTC QLQ-C30	ANOVA
	Qualitative	Focus group interviews; think-aloud Test	Thematic analysis
**Phase III: Implementation Testing, N=50**			
	Quantitative	EORTC QLQ-C30	ANOVA
	Qualitative	Additional focus group interviews if needed	Thematic analysis if needed

**Table 2:** Specific development strategies and outcome measurements used to address select domains from the Model for Assessment of Telemedicine Applications (MAST) framework. AYA: adolescent and young adult; mHealth: mobile health; EORTC QLQ-C30: European Organization for Research and Treatment of Cancer Quality of life Questionnaire Core 30.

Preceding Considerations		Challenges and required resources specified by AYAs^a^ with cancer that may be addressed by a supportive mHealth^b^ intervention
**Assessments within Seven Domains (project measurements and considerations)**		
	Health problem and app characteristic	Quality of life in AYAs with cancer and cocreated app specifically oriented to this patient population
	Safety	Patient identifying information and data security according to Danish data regulation
	Clinical Effectiveness	EORTC QLQ-C30^c^
	Patient Perspectives	Qualitative interviews and cocreation app development
	Economic Aspects	Continuous funding for app maintenance
	Organizational Aspects	App ownership (determining role of developer and hospital)
	Sociocultural, Legal, and Ethical Aspects	Data ownership and protection and informed consent of project participants
Transferability of the intervention to areas of expansion		Extension of the cocreation process may be useful for the development of future mHealth interventions. Further evaluation of transferability and areas of expansion will be discussed in future publications

In addition, participants will be asked to evaluate the app in two ways, first individually via a “Think Aloud” test and, secondly, by focus group interviews (asking questions about the cancer needs of the individual). Both the Think Aloud test and focus group interviews will be conducted on the same day in the Kræftværket day room. Participants will be asked to schedule their date for the Think Aloud test and focus group interviews during the informed consent process. The Think Aloud test and focus group interviews will be scheduled within two weeks of the 6-week app utilization period. All Think Aloud tests and focus group interviews will be performed with respect to the needs and physical condition of AYAs included in the study.

The “Think Aloud” method will be used to test the app's functionalities, as the patient is encouraged to verbally express likes, dislikes, comments, and concerns regarding the use the app [45]. This method of evaluating usability was selected as it provides both insight into the straightforward utilization of the app, as well as the user’s personal insight and opinion of app utilization [45,46]. By using the Think Aloud test, it is possible to follow the decision-making processes of app users to understand which features are seen as useful or not useful, what questions arise and what aspects of the app are or are not intuitive [46]. The Think Aloud test will be performed by the app developer.

Focus group interviews will also be utilized to evaluate the performance of the app. Participants will be interviewed about their general perception of the app, as well as how the app relates to the following topics: everyday life, community and loneliness, information resources, symptoms, existential and identity issues, and empowerment. These focus groups will be conducted by an external researcher experienced in qualitative research, according to a semistructured interview guide. Focus group interviews will be recorded, transcribed verbatim, and analyzed for content using thematic analysis [47].

Results and appropriate analysis from both the qualitative and quantitative branches of the pilot test will be discussed amongst the research group, and appropriate changes based on user feedback will be made to the app before the final project phase.

### Phase III: Implementation Testing and Quality of Life Evaluation

In the final phase, the app will be provided and utilized over 3 months by a sample of 50 AYAs aged 15-29 years whose treatment is either in progress or completed. Participants will complete the EORTC QLQ-C30 at baseline (before utilization of the app), and then repeat the EORTC QLQ-C30 via the app, after prompting, at the end of the three-month period. Quantitative data will be analyzed using one-way ANOVA test.

If a need for further qualitative analysis is identified during Phase II, additional focus group qualitative interviews using a semistructured interview guide will be performed during Phase III on an as-needed basis. If additional focus group interviews are performed, these interviews will be recorded, transcribed verbatim, and analyzed for content using Thematic Analysis [47].

### Ethical Considerations

All identifying patient information will be anonymized. Data collected from the app, including app usage and content, will only be evaluated as a whole. As such, no data within the app will be able to be seen or identified from a specific individual.

All participants will sign informed consent forms prior to participation in any study procedure. If a participant is under the age of 18, caregiver informed consent will additionally be obtained. The study has been submitted to the Danish Data Protection Agency. This protocol is to be performed in accordance with the ethical recommendations of the Helsinki declaration. Patient confidentiality will be assured, and in future publications no identifying patient information will be utilized. Alphanumeric codes will be used throughout data analysis to anonymize patients. No identifying patient information, such as name or birthdate, will be used when discussing qualitative or quantitative data. Ethical approval of qualitative studies by the regional ethics committee is not necessary in Denmark.

## Results

The development and trial of the Kræftværket app received funding February 2017 from Trygfonden, a non-profit foundation. At the time of this paper’s submission, participants had completed 3 app development workshops and 3 ad hoc meetings between September 2016 through August 2017, and the Kræftværket app was in the final stages of visual design and programming for iOS and Android. Pilot testing and initial QoL-research will take place in February 2018, and implementation and extended QoL testing is expected to begin May 2018.

## Discussion

### Principal Findings

This paper outlines a protocol for the evaluation of a user cocreated smartphone app for AYAs with cancer. While many mHealth apps exist, few have been thoroughly investigated to determine their efficacy and benefit [20] and this is particularly true for AYAs with cancer. In literature to date, there is a significant absence of apps that have been thoroughly evaluated [21,32]. It is the goal of the research team behind this protocol to address this absence with our evaluation of Kræftværket app.

Smartphone app interventions have great potential to benefit AYA oncology patients through increased access to information, symptom and status tracking, and supportive social networking. In prior studies, a lower HRQoL was associated with decreased autonomy, social support, coping abilities, and unmet information needs among AYA patients with cancer [25,48-50]. However, literature reviews focusing on HRQoL have also indicated that increased social support—such as that from family, friends, or other cancer survivors—can improve HRQoL to benefit patient outcomes [6]. Social support from other cancer survivors has been previously noted as particularly helpful in improving HRQoL [25,50,51]. Other app features such as symptom tracking diaries and similar tools also show promise in supporting AYA app users and have been reported as helpful and easy to adhere to [52]. Lastly, meeting information needs with AYA-associated resources are frequently attributed to changes in HRQoL [6,48,53] As such, the inclusion of an information database featuring verified information in a youth-friendly format is another valuable feature.

It can be hypothesized that the provision of disease and personal self-management via tracking, a supportive social system, and the availability of information will benefit HRQoL. The utilization of an app platform will add an additional level of benefit—as a mHealth intervention will allow the utilization of support resources regardless of geographic location or time [18,19].

### Limitations

This project could be limited by the population size of AYA oncology patients in Denmark, approximately 500 per year [5]. On the other hand, Denmark makes an excellent model country due to a nationalized registry of cancer patients, as well as a nationalized health care system that alleviates pre-existing burdens due to insurance and welfare systems [14,15]. It is the authors’ expectation that the results of a Danish study can provide a model situation for the development of an app for young people, and that the results and findings from this project can then be utilized in the development of future apps outside of Denmark, helping AYAs both on a national and international scale.

A second limitation of the project is the timeline. Phase III will occur over a period of 3 months. However, pending the resources attached to our current project, we believe that 3 months will be sufficient to complete pilot testing, analyze data, and make changes to the app in response to pilot participants’ feedback. Longer term follow-up is not proposed in the current timeline due to funding limitations but may be pursued at a later time. Furthermore, it will be difficult to determine whether or not the app will continue to be utilized longer-term by AYA cancer survivors after the immediate time of their illness and recovery. Further studies should be performed to determine the utilization of an app for AYA cancer patients and survivors beyond the course of three months.

### Conclusion

Many apps have attempted to address the needs of AYAs with cancer, but there are few apps that have been reviewed to a sufficient standard of scientific merit. The Kræftværket app’s analysis protocol aims to address this. The process of evaluating the app and its effect on quality of life will address the shortage of literature-backed mHealth interventions, and attempt to validate new approaches to benefitting AYA oncology patients in the digital world.

## Abbreviations

ANOVA: analysis of variance
AYA: adolescent and young adult
EORTC QLQ-C30: European Organization for Research and Treatment of Cancer Quality of life Questionnaire Core 30
HRQoL: Health Related Quality of Life
MAST: Model for Assessment of Telemedicine Applications
mHealth: mobile health
PPI: patient and public involvement
QoL: quality of life
